# Bacterial Communities in the Rhizosphere of Amilaceous Maize (*Zea mays* L.) as Assessed by Pyrosequencing

**DOI:** 10.3389/fpls.2016.01016

**Published:** 2016-07-29

**Authors:** David Correa-Galeote, Eulogio J. Bedmar, Antonio J. Fernández-González, Manuel Fernández-López, Gregorio J. Arone

**Affiliations:** ^1^Department of Soil Microbiology and Symbiotic Systems, Estación Experimental del Zaidín, Agencia Estatal Consejo Superior de Investigaciones CientíficasGranada, Spain; ^2^Department of Agricultural Sciences, National University of HuancavelicaHuancavelica, Peru

**Keywords:** maize rhizospheric soil, bacterial community, pyrosequncing, 16S rRNA gene, Andean chacras

## Abstract

Maize (*Zea mays* L.) is the staple diet of the native peasants in the Quechua region of the Peruvian Andes who continue growing it in small plots called chacras following ancestral traditions. The abundance and structure of bacterial communities associated with the roots of amilaceous maize has not been studied in Andean chacras. Accordingly, the main objective of this study was to describe the rhizospheric bacterial diversity of amilaceous maize grown either in the presence or the absence of bur clover cultivated in soils from the Quechua maize belt. Three 16S rRNA gene libraries, one corresponding to sequences of bacteria from bulk soil of a chacra maintained under fallow conditions, the second from the rhizosphere of maize-cultivated soils, and the third prepared from rhizospheric soil of maize cultivated in intercropping with bur clover were examined using pyrosequencing tags spanning the V4 and V5 hypervariable regions of the gene. A total of 26031 sequences were found that grouped into 5955 distinct operational taxonomic units which distributed in 309 genera. The numbers of OTUs in the libraries from the maize-cultivated soils were significantly higher than those found in the libraries from bulk soil. One hundred ninety seven genera were found in the bulk soil library and 234 and 203 were in those from the maize and maize/bur clover-cultivated soils. Sixteen out of the 309 genera had a relative abundance higher than 0.5% and the were (in decreasing order of abundance) Gp4, Gp6, *Flavobacterium*, Subdivision3 genera incertae sedis of the Verrucomicrobia phylum, *Gemmatimonas*, *Dechloromonas*, *Ohtaekwangia*, *Rhodoferax*, *Gaiella*, *Opitutus*, Gp7, *Spartobacteria* genera incertae sedis, *Terrimonas*, Gp5, *Steroidobacter* and *Parcubacteria* genera incertae sedis. Genera Gp4 and Gp6 of the Acidobacteria, *Gemmatimonas* and *Rhodoferax* were the most abundant in bulk soil, whereas *Flavobacterium*, *Dechloromonas* and *Ohtaekwangia* were the main genera in the rhizosphere of maize intercropped with bur clover, and Gp4, Subdivision3 genera incertae sedis of phylum Verrucomicrobia, Gp6 and *Rhodoferax* were the main genera in the rhizosphere of maize plants. Taken together, our results suggest that bur clover produces specific changes in rhizospheric bacterial diversity of amilaceous maize plants.

## Introduction

Maize (*Zea mays* L.) reached the southern Andean highlands after its domestication in Mexico some 8700 years before the present (Piperno et al., 2009; van Heerwaarden et al., 2011; Grobman et al., 2012), and is since then the staple diet of the Quechua natives. Despite 500 years of Western colonization and national modernization efforts, native peasants continue growing amilaceous maize as did their ancestors, mostly without chemical fertilization and no irrigation. Most farmers (94.3%) do not use certified seeds because the fields are planted with their own community seeds (Instituto Nacional de Estadística e Informática [INEI], 2012), which include more than 1600 entries grouped in 55 races (Manrique, 1997).

It is well established that, in addition to mineral fertilizers, which are not usually employed in the Quechua agricultural practices, N inputs to agriculture can be obtained from symbiotic nitrogen fixation by rhizobial bacteria inside root nodules of leguminous plants (Sprent, 2009), and that rhizobia associated with feed/fodder legumes contribute a substantial proportion of this fixed nitrogen (Herridge et al., 2008). In the traditional Quechua farming, amilaceous maize seeds are used for sowing at each growing season and plants grow associated with innately emerged bur clover (*Medicago hispida*, Gaernt. syn. *M. polymorpha* L.), an annual shrub of the *Fabaceae* family within the tribe *Trifolieae* which, though native to the Mediterranean basin, is distributed worldwide. In the Peruvian Andes bur clover grows up to almost 4000 m above sea level where it is also widely used as a pasture and as a cover crop, greatly contributing to the fodder units required for feeding of the national cattle. The microsymbiont from nodules of bur clover has been isolated and identified as *Ensifer medicae*, and the rhizobial-*M. hispida* symbiotic association has been suggested to be the main N source for growth of maize plants in the Andean chacras (Arone et al., 2014).

The plant rhizosphere is a complex environment where soil microbial communities play a key role in ecosystems functions and are among the most complex, diverse and important assemblages in the biosphere. Accordingly, the study of plant-associated microorganisms is of great interest for their contribution to plant nutrition, hormonal control of plant growth, disease suppression, etc. (Bastida et al., 2009). Many studies on the composition and community structure of plant rhizosphere have been performed by using culture-dependent techniques and low-resolution molecular methods such as DGGE, TGGE, PLFA and SSCP profiles of microbial communities. Although isolation of culturable bacteria is appropriate for functional analysis, this approach often shows a rather limited diversity because a high percentage of naturally occurring bacteria remains in a non-culturable state (Oliver, 2000; Torsvik et al., 2002). Culture-independent molecular methods provide additional information on the diversity of bacterial communities by analyzing and comparing a very large number of samples. Among them, the bar-coded pyrosequencing technology introduced by 454 Life Science (Margulies et al., 2005; Rothberg and Leamon, 2008) describes microbial community composition in complex habitats such as the deep sea (Sogin et al., 2006; Huse et al., 2008; Polymenakou et al., 2015), human microbiome (Sundquist et al., 2007; Dethlefsen et al., 2008; Claesson et al., 2009; Hang et al., 2014), coastal microbial mats (Bolhuis and Stal, 2011; Tytgat et al., 2014) and dental implants (Kumar et al., 2012; Dabdoub et al., 2013), and soil (Uroz et al., 2010; Mao et al., 2011; Barberán et al., 2012; Sun et al., 2014; Sengupta and Dick, 2015).

Previous studies have analyzed bacterial taxa associated with maize. Some of these studies have been focused on the culturable fraction (Rai et al., 2007; Rijavec et al., 2007; Pereira et al., 2009) and others have assessed bacterial diversity independently of culture (Schmalenberger and Tebbe, 2003; Herschkovitz et al., 2005a,b; Sanguin et al., 2006a,b; Pereira et al., 2011; Ikeda et al., 2013). New generation sequencing has also been used to analyze the diversity and heritability of the maize rhizosphere microbiome under field conditions (Peiffer et al., 2013) and the importance of rare taxa for bacterial diversity in the rizosphere of Bt- and conventional maize varieties (Dohrmann et al., 2013). Because bacterial diversity in roots of amilaceous maize grown in Andean chacras has not been studied using high throughput sequencing, the main objective of this work was to describe the abundance and structure of bacterial communities associated with the roots of amilaceous maize by pyrosequencing. Given that amilaceous maize grows together with bur clover, we hypothesized that plant cultivation type could affect abundance and composition of maize rhizospheric bacteria. To test this hypothesis, we have analyzed changes on rhizospheric bacterial diversity in the absence and the presence of bur clover.

## Materials and Methods

### Site Description and Soil Sampling

Chacras are small parcels of soil (200–10000 m^2^) used by Quechua peasants to grow maize, quinoa, wheat, potatoes, and others cereals and vegetables. Soils were taken from three chacras within the same soil plot located near Allpas (12° 50′ 27″ S, 74° 34′ 14″ W, at 3537 m above sea level), a village in the province of Acobamba (Huancavelica, Peru). At sampling time, one chacra had been cultivated with amilaceous maize (M soil) for 3 years, other with maize and bur clover (MT soil) for 5 years, and the third was bulk soil from a chacra under fallow conditions (B soil) for at least 3 years. Seeds of *Z. mays* L. morphotype Qarway were planted by mid October 2012 and soil samples collected 120 days later, when the plants were at the grain filling stage. At the second hilling stage, 75–90 days after planting, bur clover looms up without previous sowing and has to be removed by hand to maintain the chacras free of the legume. Lateral roots (∼2 mm diameter, 2–3 cm long; 5–10 cm depth) were taken from maize plants grown at four different sites within each chacra and four replicates were sampled for each site. After cleaning of the soil attached to the roots, the remaining adhering rhizospheric soil was carefully removed and pooled together to obtain 2 g. Bulk soil samples (four sites, four replicates/site; 0–5 cm depth) were taken from the chacra under fallow conditions. Soil samples were kept at -20°C until further processing. The three chacras belong to the same soil plot, which has a sandy-loam texture (62.5% sand, 30.0% silt, 7.5% clay); the average pH was 6.3 and that of the soil organic matter was 28.7 g/soil kg.

### Extraction of DNA from Soil

DNA was extracted from 250 mg of unfrozen soil as previously indicated (Correa-Galeote et al., 2013). Essentially, samples were homogenized in 1 ml of extraction buffer containing 100 mM Tris (pH 8.0), 100 mM EDTA, 100 mM NaCl, 1% (w/v) polyvinylpyrrolidone and 2% (w/v) sodium dodecyl sulfate using a 2-ml mini-bead-beater tube containing 0.5 g and 0.1 g of 106-μm- and 2-mm-diameter glass beads, respectively, for 60 s at 1600 rpm. Cell debris was eliminated by centrifugation (14000 rpm for 5 min at 4°C). Proteins were removed by treatment with 5 M sodium acetate. After treatment for 12 h with ice-cold isopropanol, nucleic acids were precipitated by centrifugation (14000 rpm for 30 min at 4°C), washed with 70% ice-cold ethanol, recentrifruged (14000 rpm for 15 min at 4°C) and air-dried for 30 min. Finally, DNA was purified using GeneClean columns (Qiagen). Quality and size of DNA were checked by electrophoresis on 1% agarose and quantified by spectrophotometry at 260 nm using a Nanodrop spectrophotometer (NanoDrop ND1000).

### Amplification and Pyrosequencing of DNA from Maize Roots

Polymerase chain reaction (PCR) amplification of the hypervariable V4–V5 regions of the 16S rRNA gene was performed over each individual DNA extraction from soils using universal primers U519F and U926R (Baker et al., 2003) joined to a multiplex identifier sequence (Binladen et al., 2007; Parameswaran et al., 2007). For each sample, amplicons were generated in several replicate PCRs using mixtures (25 μl) that contained 25 pmol of each primer, 1.8 mM MgCl_2_, 0.2 mM dNTPs, 1 × the corresponding *Taq* buffer, 1 U of *Taq* Master (5 Prime, USA) and 10 ng of the DNA template. The PCR program consisted of an initial denaturation step at 94°C for 4 min, 25 cycles of denaturation at 94°C for 15 s, primer annealing at 55°C for 45 s and extension at 72°C for 1 min, followed by a final step of heating at 72°C for 10 min. Amplicons of the same treatment were pooled to reduce per-PCR variability and purified using the ultracentrifugal filters Ultracel-100 K membranes (Amicon) according to the manufacturer’s instructions. After quantification by Nanodrop ND1000 and visualization of the DNA by agarose electrophoresis, the samples were combined in equimolar amounts and pyrosequenced in a Roche Genome Sequencer FLX system using 454 Titanium chemistry at LifeSequencing S.L. (Valencia, Spain).

### Taxonomic Assignment of Sequence Reads and Diversity Indexes

Raw sequences were processed through the Ribosomal Database Project (RDP) pyrosequencing pipeline^1^ release 11 (Cole et al., 2014). Sequences were trimmed for primers, filtered and assigned to three libraries (BG, MG and MTG containing 16S rRNA gene sequences from B, M and MT soils, respectively) according to their tags. Sequences shorter than 150 base pair, with quality scores <20 or containing any unresolved nucleotides were removed from the dataset. Chimeras were identified using the Uchime tool from FunGene database (Edgar et al., 2011) and removed from the dataset. Sequences were aligned using the Infernal alignment tool in RDP (Nawrocki et al., 2009). Aligned sequences were clustered into operational taxonomic units (OTUs) defined at 97% similarity cutoff using Complete Linkage Clustering RDP tool and their relative abundances calculated. The number of sequences in each OTU was employed to calculate the Good’s coverage index, which is considered a relative measure of how well the sequences obtained represent the entire populations (Hughes and Bohannan, 2004). The RDP Classifier, a Bayesian rRNA classifying algorithm (Wang et al., 2007), was used to assign phylogenetic groups based on sequence similarity. Matches with an RDP confidence estimate below 60% were designated as unclassified bacteria. Shannon (H′) and Simpson (S′) diversity indexes and Jaccard indexes (*J*_class_ and *J*_abund_) were used to analyze the alpha- and beta-diversity, respectively (Chao et al., 2005).

### Statistical Analyses

Relative abundances of the main genera were compared using the *G*-test (w/Yates’) + Fischer’s test in the STAMP software (Parks et al., 2014). Comparisons among the total number of sequences in the genomic libraries were done using the Newcombe–Wilson method (Newcombe, 1998; Parks and Beiko, 2010).

### Accession Numbers

Pyrosequencing reads have been sent to GenBank to obtain their under accession numbers. They are available as KX479121-KX485313, KX559454-KX570596 and KX470813-KX479120 for the genomic libraries MTG, MG and BG, respectively.

## Results

### Pyrosequencing and Sequence Analysis

**FIGURE 1:**
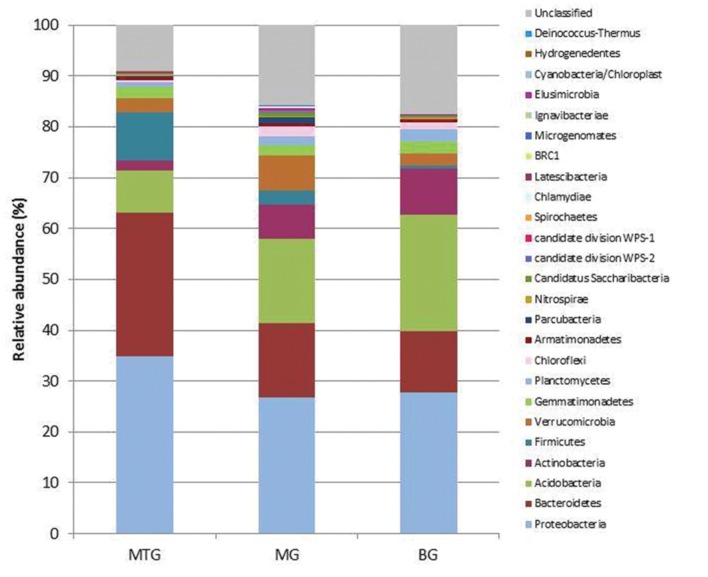
**Relative abundance of bacterial phyla in genomic libraries from bulk soil (BG) and rhizospheric soil of maize (MG) and maize intercropped with clover (MTG) grown in Andean chacras**.

A total of 38786 sequences were obtained from the three 16S rDNA samples sent to pyrosequencing, of which 26031 were retained after filtering and removing chimeras. The mean number of total retained sequences per library was 8677, ranging from 6286 to 11387. Average length of retained sequences was 374 ± 5 base pair (mean ± SD). All the sequences aligned correctly in the expected position of the 16S rDNA sequence of *Escherichia coli* and were grouped at 97% similarity in 5955 distinct OTUs, of which 1846, 3287 and 2318 were found in MTG, MG and BG libraries, respectively (**Table 1**), which also contained 575 (9.15%), 1793 (15.75%), and 1460 (17.47%) unclassified sequences, respectively (**Table 2**; **Figure 1**). The remaining valid sequences distributed into 21, 23, and 21 phyla, respectively (**Table 2**), of which phyla Proteobacteria, Bacteroidetes, Acidobacteria, Actinobacteria, Firmicutes, Verrucomicrobia, Gemmatimonadetes, Planctomycetes, Chloroflexi, Armatimonadetes, Parcubacteria, Nitrospirae, Candidatus Saccharibacteria, candidate division WPS-2, candidate division WPS-1, Latescibacteria, BRC1 and Microgenomates (in decreasing abundance) were found in all the three libraries (**Figure 1**). Phyla Spirochaetes and Chlamydiae were found in MTG and MG and so were Ignavibacteriae and Cyanobacteria in libraries MG and BG, respectively (**Figure 1**). Hydrogenedentes was detected only in MTG, Deinococcus–Thermus in MG and Elusimicrobia in BG (**Figure 1**). Numbers of classes, orders, families and genera are also shown in **Table 2**.

**Table 1:** Number of OTUs, values of Good’s coverage index, and Shannon and Simpson biodiversity indexes in genomic libraries from bulk soil (BG) and rhizospheric soil of maize (MG) and maize intercropped with clover (MTG) grown in Andean chacras.

Diversity index	MTG	MG	BG
Number of OTUs	1846	3287	2318
Good’s coverage	85.58	84.92	86.44
Shannon	6.55	7.21	7.06
Simpson	0.0056	0.0031	0.0016


**Table 2:** Number of taxa and of 16S rRNA sequences in genomic libraries from bulk soil (BG) and rhizospheric soil of maize (MG) and maize intercropped with clover (MTG) grown in Andean chacras.

	Genomic Libraries					
	
	MTG		MG		BG	
			
	Number of taxa	Number of sequences (%)	Number of taxa	Number of sequences (%)	Number of taxa	Number of sequences (%)
Phylum	21	5711 (90.85)	23	9594 (84.26)	21	6898 (82.53)
Class	41	5066 (80.59)	44	8390 (73.68)	42	8358 (75.93)
Order	43	3933 (62.57)	51	5136 (45.10)	45	3391 (39.97)
Family	43	3607 (57.38)	51	4141 (36.37)	45	2620 (31.35)
Genus	203	3131 (49.81)	234	5650 (49.62)	197	4003 (47.89)
Unclassified sequences		575 (9.15)		1793 (15.75)		1460 (17.47)


*The percentage of sequences in relation with the total number of sequences is shown in brackets.*

### Coverage and Diversity Indexes

With values of the Good’s coverage index higher than 80% at 90% confidence interval (**Table 1**), the Shannon index of 5.13 for OTUs in the MTG clone library was lower than those of 7.06 and 7.21 for the FG and MG libraries, respectively. The Simpson index, however, showed clear differences among the OTUs in each library, with values of 0.0016, 0.0031 and 0.0056 for the MTG, MG and BG clone libraries, respectively (**Table 1**).

Sequences in library BG distributed in 197 genera, a value lower than those of 203 and 234 found in libraries MTG and MG, respectively (**Table 2**). Of the total number of genera, the BG library shared 132 and 155 with the MTG and MG libraries, respectively, and libraries MTG and MG had 159 genera in common (**Table 3**). Values of the *J_class_* index were similar between the BG and MG libraries (0.56) and the MTR and MG libraries (0.57), values that were higher than that (0.49) between the MTG and BG libraries (**Table 3**). The *J_abund_* indexes for the multiple comparisons among genomic libraries were similar (**Table 3**).

**Table 3:** Number of shared genera among genomic libraries, and Jaccard similarity indexes using presence/absence (*J*_class_) and relative abundances (*J*_abund_) of the genera in libraries from bulk soil (BG) and rhizospheric soil of maize (MG) and maize intercropped with clover (MTG) grown in Andean chacras.

	Number of shared genera	*J*_class_	*J*_abund_
MTG-BG	132	0.49	0.94
MG-BG	155	0.56	0.92
MTG-MG	159	0.57	0.98


### Taxonomic Composition and Statistical Analyses

**FIGURE 2:**
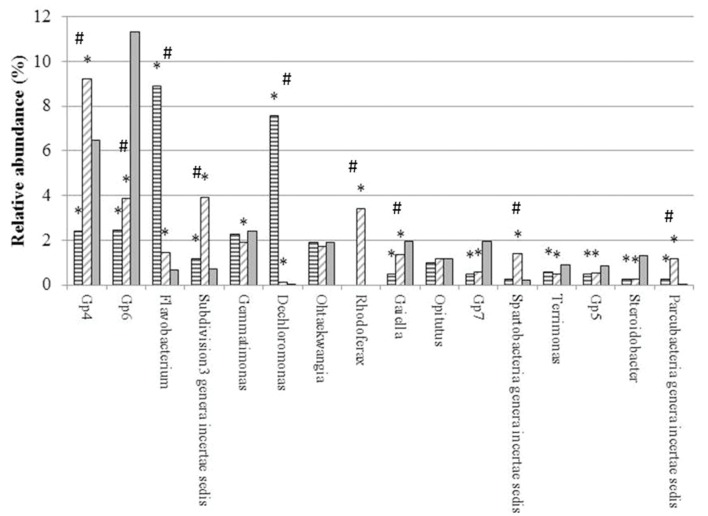
**Relative abundance (%) of main genera in genomic libraries from bulk soil (BG, solid gray) and rhizospheric soil of maize (MG, horizontal gray lines) and maize intercropped with clover (MTG, diagonal gray lines) grown in Andean chacras.** Relative abundances were compared using the combined G + Fischer tests (α ≤ 0.05). ^∗^indicates statistically differences between the sequences in the MTG and MG libraries as compared to the BG library; # indicates statistically differences between the sequences in the MTG and MG libraries.

From the total 26031 sequences, only 12784 could be assigned to 309 different genera (**Supplementary Table S1**). Sixteen (16) out of the 309 genera had a relative abundance higher than 0.5%, and represented the 64.23% of the total number of sequences classified at the genus level. Altogether, these genera were (in decreasing order of abundance) Gp4, Gp6, *Flavobacterium*, Subdivision3 genera incertae sedis of the Verrucomicrobia phylum, *Gemmatimonas*, *Dechloromonas*, *Ohtaekwangia*, *Rhodoferax*, *Gaiella*, *Opitutus*, Gp7, *Spartobacteria* genera incertae sedis, *Terrimonas*, Gp5, *Steroidobacter* and *Parcubacteria* genera incertae sedis (**Supplementary Table S1**) (**Figure 2**).

Relative abundances of genera *Flavobacterium*, Subdivision3 genera incertae sedis of the Verrucomicrobia phylum, *Dechloromonas* and *Parcubacteria* in each the MTG and MG libraries were statistically higher than those in the BG library. In contrast, the number of sequences of genera Gp6, *Gaiella*, Gp7, *Terrimonas*, Gp5 and *Steroidobacter* in the MTG and MG libraries were lower than those in the BG library.

Numbers of sequences of genera *Flavobacterium* and *Dechloromonas* in the MTG library were statistically higher than those in the MG library (**Figure 2**). On the other hand, the relative abundance of genera Gp4, Gp6, Subdivision3 genera incertae sedis of phylum Verrucomicrobia, *Rhodoferax*, *Gaiella* and *Spartobacteria* in MTG were significantly lower than those in the MG library (**Figure 2**). Differences in the relative abundance of sequences corresponding to genera *Gemmatimonas*, *Ohtaekwangia*, *Opitutus*, Gp7, *Terrimonas*, Gp5, *Steroidobacter* and *Parcubacteria* were not found between the MTG and MG libraries (**Figure 2**).

## Discussion

Using 454 next generation sequencing we assessed the composition and abundance of bacterial rhizospheric communities of amilaceous maize plants grown in Andean chacras following Quechua traditional practices. The total number of genera found in our genomic libraries were higher than those obtained in previous studies based on culture-dependent and culture-independent methods (Gomes et al., 2001; Schmalenberger and Tebbe, 2002; Baumgarte and Tebbe, 2005; Brusetti et al., 2005; Aira et al., 2010; Miethling-Graff et al., 2010; Pereira et al., 2011; García-Salamanca et al., 2013; Li et al., 2014), and similar to those found in the rhizosphere of different maize varieties after pyrosequencing (Dohrmann et al., 2013).

Altogether, a 12.71% of the total sequences found inside roots corresponded to unclassified bacteria, which indicates the presence of hitherto uncultured bacterial groups. A high percentage of unclassified sequences have also been reported in the rhizosphere of maize plants when pyrosequencing was used to study bacterial biodiversity (Dohrmann et al., 2013).

A variety of bacteria have been reported to be rhizospheric, among them mostly Proteobacteria, Firmicutes, Actinobacteria, Acidobacteria and Bacteroidetes (Sharma et al., 2005; DeAngelis et al., 2009; Peiffer et al., 2013; Uroz et al., 2013). In our study, regardless of soil provenance, members of phylum Proteobacteria were the most abundant (29.10%) followed by those of Bacteroidetes (17.03%), Acidobacteria (16.62%), Actinobacteria (6.40%), Verrucomicrobia (4.48%) and Firmicutes (3.55%) (**Figure 1**). Although the amount of bacterial phyla found in the three genomic libraries was similar (**Table 2**), the number of OTUs and values of the Shannon and Simpson indexes for the MTG library were lower than those for the BG library (**Table 1**), which suggests that intercropping reduced richness of the community. These results agree with those previously published that show that microbial diversity in maize rhizospheric soils was lower than in bulk soils (García-Salamanca et al., 2013; Peiffer et al., 2013). This reduction can be explained if one considers that maize plants intercropped with bur clover somehow select from the bulk soil those bacteria able to promote plant growth and development; e.g., due to the symbiosis with *E. medicae* (Arone et al., 2014) free-living *N*_2_ bacteria would not be required for plant growth. Our results also show that diversity in the MG library was higher than that in the BG library (**Table 1**). It is possible that the absence of bur clover promts the plants to increase bacterial diversity around the roots to improve their nutrition. Alternatively, it is also possible that more time is required for the maize crop to become well established in soils, which, in turn, could alter the soil microbiota. On the other hand, clear dominance of specific rhizospheric bacteria was not found, and multiple comparisons analyses of the values of the β-diversity indexes indicated the three genomic libraries have a similar bacterial community core. The absence of specific phylogenetic groups between bulk and rhizospheric soil from an oak forest after pyrosequencing of the 16S rRNA gene has been reported (Uroz et al., 2010).

Plants influence the abundance and composition of the bacterial rhizospheric community by secreting a variety of compounds through their roots into the surrounding soil to feed and manipulate the microbes that live there (Johnston-Monje et al., 2016). If one considers the bulk soil of the chacras as the reservoir of microbial communities, whereas maize increased relative abundance of genera Gp4, *Flavobacterium*, Subdivision3 genera incertae sedis of the Verrucomicrobia phylum, *Dechloromonas*, *Rhodoferax*, *Spartobacteria* and *Parcubacteria* incertae sedis, the maize-bur clover consortium increased relative abundance of *Flavobacterium*, Subdivision3 genera incertae sedis of the Verrucomicrobia phylum, *Dechloromonas* and *Parcubacteria* incertae sedis.

It is well established that plant genotype plays a significant role in shaping plant-associated microbial communities and in determining the biological outcome of such associations (Smith and Goodman, 1999; Ding et al., 2013). In this sense, 16S rRNA-based pyrosequencing studies on microbial diversity from rhizosphere of different maize genotypes have shown that variation in microbial diversity could be attributed to host genetics (Bouffaud et al., 2012; Peiffer et al., 2013). Because seeds of amilaceous maize used for planting were the same and the environmental conditions (including soil type and physicochemical properties), were, also, the same for the three chacras, our results suggest that the plant could be a main factor controlling bacterial diversity in their rhizosphere, whether alone (maize) or in consortium with other plants (maize and bur clover). Since richness of bacteria colonizing maize roots was based on pyrosequencing, it is not known whether the detection of bacteria based on DNA signature alone represent active microbes that are interacting with the host plant.

## Author Contributions

Conceived and designed the experiments: DC-G, EJB, GJA. Performed the experiments: DC-G, GJA. Analyzed the data: DC-G, EJB. Contributed reagents/materials/analysis tools: AJF-G, MF-L. Improve the paper: AJF-G, MF-L, GJA. Wrote the paper: DC-G, EJB.

## Conflict of Interest Statement

The authors declare that the research was conducted in the absence of any commercial or financial relationships that could be construed as a potential conflict of interest.

## Footnotes

1 http://pyro.cme.msu.edu
