# Supplementary material for: Bacterial Communities in the Rhizosphere of Amilaceous Maize (Zea mays L.) as Assessed by Pyrosequencing
Source: Front Plant Sci. 2016 Jul 29;7:1016. doi: 10.3389/fpls.2016.01016 (PMC4966391; doi:10.3389/fpls.2016.01016)
Supplement: Supplementary file 1 [file Table_1.DOCX]

Table 1S. Taxonomic classification and number of bacterial sequences in genomic libraries from bulk soil (BG) and rhizospheric soil of maize (MG) and maize intercropped with clover (MTG) grown in Andean chacras.

| Phylum | Class | Order | Family | Genus | Number of bacterial sequences | | |
| --- | --- | --- | --- | --- | --- | --- | --- |
|  |  |  |  |  | MTG | MG | BG |
| Acidobacteria | Acidobacteria Gp4 |  |  | Gp4 | 151 | 1046 | 540 |
| Acidoobacteria | Acidobacteria Gp6 |  |  | Gp6 | 152 | 435 | 945 |
| Bacteroidetes | Flavobacteriia | Flavobacteriales | Flavobacteriaceae | *Flavobacterium* | 558 | 162 | 57 |
| Verrucomicrobia | Subdivision3 |  |  | Subdivision3 genera  incertae sedis | 72 | 445 | 59 |
| Gemmatimonadetes | Gemmatimonadetes | Gemmatimonadales | Gemmatimonadaceae | *Gemmatimonas* | 141 | 214 | 202 |
| Proteobacteria | Betaproteobacteria | Rhodocyclales | Rhodocyclaceae | *Dechloromonas* | 473 | 12 | 1 |
| Bacteroidetes | Bacteroidetes incertae sedis |  |  | *Ohtaekwangia* | 118 | 195 | 157 |
| Proteobacteria | Betaproteobacteria | Burkholderiales | Comamonadaceae | *Rhodoferax* | 0 | 387 | 0 |
| Actinobacteria | Actinobacteria | Gaiellales | Gaiellaceae | *Gaiella* | 30 | 151 | 164 |
| Verrucomicrobia | Opitutae | Opitutales | Opitutaceae | *Opitutus* | 61 | 132 | 98 |
| Acidobacteria | Acidobacteria Gp7 |  |  | Gp7 | 28 | 62 | 162 |
| Verrucomicrobia | Spartobacteria |  |  | Spartobacteria genera  incertae sedis | 15 | 156 | 17 |
| Bacteroidetes | Sphingobacteriia | Sphingobacteriales | Chitinophagaceae | *Terrimonas* | 36 | 50 | 75 |
| Acidobacteria | Acidobacteria Gp5 |  |  | Gp5 | 30 | 56 | 72 |
| Proteobacteria | Gammaproteobacteria | Xanthomonadales | Sinobacteraceae | *Steroidobacter* | 15 | 24 | 109 |
| Parcubacteria |  |  |  | *Parcubacteria* genera  incertae sedis | 16 | 128 | 2 |
| Proteobacteria | Alphaproteobacteria | Sphingomonadales | Sphingomonadaceae | *Sphingomonas* | 24 | 55 | 47 |
| Proteobacteria | Gammaproteobacteria | Xanthomonadales | Xanthomonadaceae | *Arenimonas* | 88 | 15 | 22 |
| Acidobacteria | Acidobacteria Gp3 |  |  | Gp3 | 23 | 38 | 61 |
| Proteobacteria | Betaproteobacteria | Burkholderiales | Comamonadaceae | *Acidovorax* | 102 | 4 | 8 |
| Planctomycetes | Planctomycetia | Planctomycetales | Planctomycetaceae | *Pirellula* | 8 | 51 | 53 |
| Firmicutes | Bacilli | Bacillales | Bacillaceae 1 | *Bacillus* | 40 | 59 | 10 |
| Bacteroidetes | Sphingobacteriia | Sphingobacteriales | Chitinophagaceae | *Ferruginibacter* | 51 | 30 | 16 |
| Candidatus Saccharibacteria |  |  |  | *Saccharibacteria* genera  incertae sedis | 5 | 83 | 8 |
| Nitrospirae | Nitrospira | Nitrospirales | Nitrospiraceae | *Nitrospira* | 22 | 20 | 43 |
| Firmicutes | Clostridia | Clostridiales | Ruminococcaceae | *Clostridium* III | 72 | 11 | 0 |
| Proteobacteria | Alphaproteobacteria | Caulobacterales | Caulobacteraceae | *Phenylobacterium* | 17 | 35 | 28 |
| Proteobacteria | Deltaproteobacteria | Myxococcales | Haliangiaceae | *Haliangium* | 8 | 23 | 49 |
| candidate division WPS-2 |  |  |  | WPS-2 genera | 8 | 57 | 12 |
|  |  |  |  | incertae sedis |  |  |  |
| Bacteroidetes | Sphingobacteriia | Sphingobacteriales | Sphingobacteriaceae | *Mucilaginibacter* | 12 | 54 | 5 |
| Proteobacteria | Gammaproteobacteria | Pseudomonadales | Pseudomonadaceae | *Pseudomonas* | 24 | 42 | 2 |
| Bacteroidetes | Sphingobacteriia | Sphingobacteriales | Sphingobacteriaceae | *Pedobacter* | 13 | 29 | 25 |
| Proteobacteria | Alphaproteobacteria | Rhizobiales | Bradyrhizobiaceae | *Bradyrhizobium* | 11 | 40 | 14 |
| Actinobacteria | Actinobacteria | Actinomycetales | Nocardioidaceae | *Nocardioides* | 10 | 16 | 38 |
| Actinobacteria | Actinobacteria | Solirubrobacterales | Solirubrobacteraceae | *Solirubrobacter* | 7 | 26 | 31 |
| Armatimonadetes |  |  |  | *Armatimonadetes* gp4 | 8 | 27 | 28 |
| Acidobacteria | Acidobacteria Gp10 |  |  | Gp10 | 5 | 30 | 26 |
| Proteobacteria | Deltaproteobacteria | Desulfuromonadales | Geobacteraceae | *Geobacter* | 26 | 32 | 1 |
| Proteobacteria | Alphaproteobacteria | Rhizobiales | Rhizobiaceae | *Rhizobium* | 4 | 44 | 11 |
| Proteobacteria | Betaproteobacteria | Burkholderiales | Comamonadaceae | *Variovorax* | 19 | 25 | 9 |
| Bacteroidetes | Sphingobacteriia | Sphingobacteriales | Chitinophagaceae | *Flavisolibacter* | 12 | 27 | 14 |
| Bacteroidetes | Cytophagia | Cytophagales | Cytophagaceae | *Adhaeribacter* | 13 | 3 | 37 |
| Actinobacteria | Actinobacteria | Rubrobacterales | Rubrobacteraceae | *Rubrobacter* | 0 | 48 | 5 |
| Proteobacteria | Betaproteobacteria | Burkholderiales | Oxalobacteraceae | *Duganella* | 19 | 23 | 9 |
| Armatimonadetes | Chthonomonadetes | Chthonomonadales | Chthonomonadaceae | *Chthonomonas*/  *Armatimonadetes* gp3 | 13 | 29 | 9 |
| Acidobacteria | Acidobacteria Gp17 |  |  | Gp17 | 1 | 20 | 29 |
| Planctomycetes | Planctomycetia | Planctomycetales | Planctomycetaceae | *Planctomyces* | 12 | 15 | 22 |
| Actinobacteria | Actinobacteria | Actinomycetales | Streptomycetaceae | *Streptomyces* | 1 | 16 | 32 |
| Acidobacteria | Acidobacteria Gp1 |  |  | Gp1 | 9 | 39 | 0 |
| Proteobacteria | Gammaproteobacteria | Xanthomonadales | Xanthomonadaceae | *Lysobacter* | 6 | 11 | 31 |
| candidate division WPS-1 |  |  |  | WPS-1 genera | 5 | 30 | 10 |
|  |  |  |  | incertae sedis |  |  |  |
| Firmicutes | Clostridia | Clostridiales | Gracilibacteraceae | *Gracilibacter* | 22 | 22 | 0 |
| Proteobacteria | Alphaproteobacteria | Sphingomonadales | Sphingomonadaceae | *Novosphingobium* | 8 | 20 | 13 |
| Proteobacteria | Alphaproteobacteria | Rhodospirillales | Rhodospirillaceae | *Dongia* | 11 | 14 | 15 |
| Bacteroidetes | Flavobacteriia | Flavobacteriales | Flavobacteriaceae | *Chryseobacterium* | 1 | 38 | 1 |
| Proteobacteria | Alphaproteobacteria | Rhizobiales | Hyphomicrobiaceae | *Devosia* | 10 | 11 | 18 |
| Bacteroidetes | Sphingobacteriia | Sphingobacteriales | Chitinophagaceae | *Segetibacter* | 3 | 28 | 5 |
| Proteobacteria | Betaproteobacteria | Burkholderiales | Oxalobacteraceae | *Massilia* | 25 | 3 | 6 |
| Actinobacteria | Actinobacteria | Actinomycetales | Pseudonocardiaceae | *Pseudonocardia* | 2 | 18 | 14 |
| Bacteroidetes | Sphingobacteriia | Sphingobacteriales | Chitinophagaceae | *Chitinophaga* | 6 | 6 | 22 |
| Proteobacteria | Deltaproteobacteria | Myxococcales | Cystobacteraceae | *Anaeromyxobacter* | 1 | 23 | 9 |
| Proteobacteria | Betaproteobacteria | Burkholderiales | Comamonadaceae | *Hydrogenophaga* | 0 | 32 | 0 |
| Actinobacteria | Actinobacteria | Actinomycetales | Nocardioidaceae | *Aeromicrobium* | 1 | 10 | 20 |
| Proteobacteria | Gammaproteobacteria | Legionellales | Legionellaceae | *Legionella* | 24 | 3 | 4 |
| Firmicutes | Clostridia | Clostridiales | Clostridiaceae 1 | *Clostridium* sensu stricto | 12 | 16 | 1 |
| Proteobacteria | Betaproteobacteria | Burkholderiales | Oxalobacteraceae | *Undibacterium* | 5 | 23 | 1 |
| Bacteroidetes | Flavobacteriia | Flavobacteriales | Cryomorphaceae | *Fluviicola* | 22 | 4 | 2 |
| Proteobacteria | Gammaproteobacteria | Legionellales | Coxiellaceae | *Aquicella* | 9 | 16 | 3 |
| Planctomycetes | Planctomycetia | Planctomycetales | Planctomycetaceae | *Zavarzinella* | 6 | 15 | 6 |
| Bacteroidetes | Sphingobacteriia | Sphingobacteriales | Chitinophagaceae | *Lacibacter* | 11 | 5 | 10 |
| Proteobacteria | Betaproteobacteria | Methylophilales | Methylophilaceae | *Methylophilus* | 18 | 7 | 0 |
| Acidobacteria | Acidobacteria Gp4 |  |  | *Blastocatella* | 2 | 15 | 8 |
| Proteobacteria | Betaproteobacteria | Burkholderiales | Comamonadaceae | *Roseateles* | 8 | 8 | 8 |
| Bacteroidetes | Sphingobacteriia | Sphingobacteriales | Chitinophagaceae | *Sediminibacterium* | 13 | 9 | 1 |
| Acidobacteria | Acidobacteria Gp25 |  |  | Gp25 | 1 | 13 | 9 |
| Proteobacteria | Alphaproteobacteria | Rhizobiales | Phyllobacteriaceae | *Mesorhizobium* | 5 | 6 | 12 |
| Proteobacteria | Gammaproteobacteria | Xanthomonadales | Xanthomonadaceae | *Panacagrimonas* | 0 | 3 | 20 |
| Firmicutes | Clostridia | Clostridiales | Clostridiales Incertae Sedis XIII | *Anaerovorax* | 17 | 5 | 0 |
| Actinobacteria | Actinobacteria | Actinomycetales | Nocardioidaceae | *Marmoricola* | 3 | 9 | 10 |
| Acidobacteria | Acidobacteria Gp1 |  |  | *Candidatus* Koribacter | 7 | 13 | 1 |
| Proteobacteria | Deltaproteobacteria | Bdellovibrionales | Bdellovibrionaceae | *Bdellovibrio* | 5 | 15 | 1 |
| Bacteroidetes | Sphingobacteriia | Sphingobacteriales | Chitinophagaceae | *Flavitalea* | 3 | 8 | 10 |
| Latescibacteria |  |  |  | *Latescibacteria* genera  incertae sedis | 4 | 6 | 10 |
| Proteobacteria | Betaproteobacteria | Burkholderiales | Oxalobacteraceae | *Janthinobacterium* | 6 | 14 | 0 |
| Actinobacteria | Actinobacteria | Actinomycetales | Micrococcaceae | *Arthrobacter* | 2 | 12 | 5 |
| Planctomycetes | Planctomycetia | Planctomycetales | Planctomycetaceae | *Gemmata* | 1 | 10 | 7 |
| Actinobacteria | Actinobacteria | Actinomycetales | Mycobacteriaceae | *Mycobacterium* | 1 | 9 | 8 |
| Actinobacteria | Actinobacteria | Actinomycetales | Nocardioidaceae | *Kribbella* | 2 | 7 | 9 |
| Actinobacteria | Actinobacteria | Actinomycetales | Microbacteriaceae | *Agromyces* | 2 | 3 | 13 |
| Proteobacteria | Alphaproteobacteria | Rhizobiales | Hyphomicrobiaceae | *Pedomicrobium* | 3 | 3 | 12 |
| Acidobacteria | Acidobacteria Gp3 |  |  | *Candidatus* Solibacter | 3 | 14 | 0 |
| Proteobacteria | Alphaproteobacteria | Caulobacterales | Caulobacteraceae | *Asticcacaulis* | 5 | 6 | 6 |
| Acidobacteria | Acidobacteria Gp18 |  |  | Gp18 | 0 | 0 | 16 |
| Actinobacteria | Actinobacteria | Acidimicrobiales | Acidimicrobineae incertae sedis | *Aciditerrimonas* | 3 | 6 | 7 |
| Bacteroidetes | Bacteroidia | Bacteroidales |  | *Sunxiuqinia* | 5 | 10 | 0 |
| Proteobacteria | Alphaproteobacteria | Rhodospirillales | Rhodospirillaceae | *Skermanella* | 2 | 3 | 10 |
| Chloroflexi | Anaerolineae | Anaerolineales | Anaerolineaceae | *Longilinea* | 3 | 4 | 8 |
| Microgenomates |  |  |  | *Microgenomates* genera  incertae sedis | 1 | 9 | 5 |
| Proteobacteria | Alphaproteobacteria | Caulobacterales | Caulobacteraceae | *Caulobacter* | 3 | 2 | 9 |
| Firmicutes | Clostridia | Clostridiales | Peptococcaceae 1 | *Desulfosporosinus* | 7 | 7 | 0 |
| Proteobacteria | Alphaproteobacteria | Sphingomonadales | Sphingomonadaceae | *Sphingobium* | 8 | 6 | 0 |
| Chloroflexi | Caldilineae | Caldilineales | Caldilineaceae | *Litorilinea* | 0 | 6 | 8 |
| Proteobacteria | Betaproteobacteria | Burkholderiales | Comamonadaceae | *Pelomonas* | 3 | 9 | 1 |
| Armatimonadetes | Armatimonadia | Armatimonadales | Armatimonadaceae | *Armatimonas*/  *Armatimonadetes* gp1 | 3 | 8 | 2 |
| Planctomycetes | Planctomycetia | Planctomycetales | Planctomycetaceae | *Schlesneria* | 5 | 4 | 4 |
| Chloroflexi | Chloroflexia | Herpetosiphonales | Herpetosiphonaceae | *Herpetosiphon* | 1 | 6 | 6 |
| Actinobacteria | Actinobacteria | Actinomycetales | Microbacteriaceae | *Microbacterium* | 1 | 7 | 4 |
| Bacteroidetes | Cytophagia | Cytophagales | Cytophagaceae | *Cytophaga* | 7 | 3 | 2 |
| Actinobacteria | Actinobacteria | Actinomycetales | Geodermatophilaceae | *Blastococcus* | 1 | 2 | 9 |
| Chloroflexi | Anaerolineae | Anaerolineales | Anaerolineaceae | *Bellilinea* | 1 | 11 | 0 |
| Proteobacteria | Alphaproteobacteria | Rhodospirillales | Rhodospirillaceae | *Magnetospirillum* | 9 | 2 | 0 |
| BRC1 |  |  |  | BRC1 genera | 4 | 5 | 2 |
|  |  |  |  | incertae sedis |  |  |  |
| Proteobacteria | Deltaproteobacteria | Myxococcales | Cystobacteraceae | *Cystobacter* | 4 | 3 | 4 |
| Acidobacteria | Acidobacteria Gp22 |  |  | Gp22 | 7 | 1 | 3 |
| Actinobacteria | Actinobacteria | Actinomycetales | Propionibacteriaceae | *Microlunatus* | 2 | 7 | 2 |
| Chloroflexi | Thermomicrobia | Sphaerobacterales | Sphaerobacteraceae | *Sphaerobacter* | 1 | 4 | 6 |
| Bacteroidetes | Sphingobacteriia | Sphingobacteriales | Chitinophagaceae | *Niastella* | 2 | 2 | 7 |
| Bacteroidetes | Cytophagia | Cytophagales | Cytophagaceae | *Dyadobacter* | 0 | 5 | 6 |
| Actinobacteria | Actinobacteria | Actinomycetales | Pseudonocardiaceae | *Actinophytocola* | 0 | 6 | 5 |
| Proteobacteria | Betaproteobacteria | Burkholderiales | Comamonadaceae | *Polaromonas* | 4 | 4 | 2 |
| Firmicutes | Negativicutes | Selenomonadales | Veillonellaceae | *Pelosinus* | 5 | 5 | 0 |
| Proteobacteria | Alphaproteobacteria | Rhizobiales | Methylobacteriaceae | *Microvirga* | 2 | 1 | 7 |
| Spirochaetes | Spirochaetia | Spirochaetales | Spirochaetaceae | *Spirochaeta* | 2 | 8 | 0 |
| Proteobacteria | Alphaproteobacteria | Rhizobiales | Xanthobacteraceae | *Pseudolabrys* | 1 | 7 | 2 |
| Acidobacteria | Acidobacteria Gp11 |  |  | Gp11 | 1 | 1 | 8 |
| Proteobacteria | Deltaproteobacteria | Bdellovibrionales | Bacteriovoracaceae | *Bacteriovorax* | 7 | 2 | 0 |
| Armatimonadetes |  |  |  | *Armatimonadetes* gp5 | 1 | 8 | 0 |
| Proteobacteria | Alphaproteobacteria | Rhizobiales | Hyphomicrobiaceae | *Hyphomicrobium* | 3 | 1 | 4 |
| Proteobacteria | Alphaproteobacteria | Rhizobiales | Rhizobiales incertae sedis | *Vasilyevaea* | 1 | 2 | 5 |
| Proteobacteria | Betaproteobacteria | Burkholderiales | Comamonadaceae | *Delftia* | 2 | 6 | 0 |
| Actinobacteria | Actinobacteria | Actinomycetales | Pseudonocardiaceae | *Lentzea* | 0 | 0 | 8 |
| Actinobacteria | Actinobacteria | Actinomycetales | Micromonosporaceae | *Micromonospora* | 0 | 7 | 1 |
| Proteobacteria | Betaproteobacteria | Burkholderiales | Comamonadaceae | *Albidiferax* | 5 | 2 | 0 |
| Firmicutes | Clostridia | Clostridiales | Clostridiaceae 1 | *Fervidicella* | 6 | 1 | 0 |
| Proteobacteria | Betaproteobacteria | Burkholderiales | Oxalobacteraceae | *Noviherbaspirillum* | 3 | 3 | 1 |
| Proteobacteria | Gammaproteobacteria | Gammaproteobacteria incertae sedis |  | *Solimonas* | 0 | 5 | 2 |
| Proteobacteria | Deltaproteobacteria | Myxococcales | Nannocystaceae | *Enhygromyxa* | 3 | 2 | 2 |
| Proteobacteria | Alphaproteobacteria | Caulobacterales | Caulobacteraceae | *Brevundimonas* | 1 | 0 | 6 |
| Actinobacteria | Actinobacteria | Solirubrobacterales | Conexibacteraceae | *Conexibacter* | 1 | 5 | 1 |
| Proteobacteria | Gammaproteobacteria | Xanthomonadales | Xanthomonadaceae | *Rhodanobacter* | 6 | 1 | 0 |
| Actinobacteria | Actinobacteria | Actinomycetales | Pseudonocardiaceae | *Amycolatopsis* | 0 | 7 | 0 |
| Proteobacteria | Alphaproteobacteria | Rhodospirillales | Rhodospirillaceae | *Azospirillum* | 3 | 3 | 0 |
| Proteobacteria | Alphaproteobacteria | Alphaproteobacteria incertae sedis |  | *Rhizomicrobium* | 3 | 2 | 1 |
| Proteobacteria | Alphaproteobacteria | Rhizobiales | Methylobacteriaceae | *Methylobacterium* | 3 | 0 | 3 |
| Proteobacteria | Alphaproteobacteria | Rhizobiales | Hyphomicrobiaceae | *Rhodoplanes* | 0 | 4 | 2 |
| Proteobacteria | Gammaproteobacteria | Xanthomonadales | Xanthomonadaceae | *Stenotrophomonas* | 3 | 2 | 1 |
| Proteobacteria | Deltaproteobacteria | Bdellovibrionales | Bdellovibrionaceae | *Vampirovibrio* | 0 | 4 | 2 |
| Actinobacteria | Actinobacteria | Actinomycetales | Nocardiaceae | *Nocardia* | 0 | 3 | 3 |
| Actinobacteria | Actinobacteria | Actinomycetales | Micromonosporaceae | *Dactylosporangium* | 0 | 5 | 1 |
| Firmicutes | Clostridia | Clostridiales | Ruminococcaceae | *Sporobacter* | 2 | 3 | 0 |
| Firmicutes | Clostridia | Clostridiales | Lachnospiraceae | *Clostridium* XlVa | 4 | 1 | 0 |
| Proteobacteria | Betaproteobacteria | Burkholderiales | Burkholderiaceae | *Burkholderia* | 1 | 2 | 2 |
| Actinobacteria | Actinobacteria | Actinomycetales | Intrasporangiaceae | *Phycicoccus* | 0 | 1 | 4 |
| Ignavibacteriae | Ignavibacteria | Ignavibacteriales | Ignavibacteriaceae | *Ignavibacterium* | 0 | 4 | 1 |
| Planctomycetes | Planctomycetia | Planctomycetales | Planctomycetaceae | *Blastopirellula* | 0 | 0 | 5 |
| Firmicutes | Bacilli | Bacillales | Paenibacillaceae 1 | *Paenibacillus* | 1 | 2 | 2 |
| Proteobacteria | Betaproteobacteria | Burkholderiales | Burkholderiales incertae sedis | *Aquabacterium* | 0 | 5 | 0 |
| Actinobacteria | Actinobacteria | Actinomycetales | Geodermatophilaceae | *Geodermatophilus* | 1 | 1 | 3 |
| Proteobacteria | Betaproteobacteria | Nitrosomonadales | Nitrosomonadaceae | *Nitrosospira* | 2 | 0 | 3 |
| Proteobacteria | Gammaproteobacteria | Xanthomonadales | Xanthomonadaceae | *Luteimonas* | 1 | 0 | 4 |
| Actinobacteria | Actinobacteria | Actinomycetales | Propionibacteriaceae | *Propionibacterium* | 0 | 2 | 3 |
| Actinobacteria | Actinobacteria | Actinomycetales | Sporichthyaceae | *Sporichthya* | 0 | 3 | 2 |
| Actinobacteria | Actinobacteria | Actinomycetales | Thermomonosporaceae | *Actinomadura* | 0 | 4 | 1 |
| Proteobacteria | Gammaproteobacteria | Pseudomonadales | Pseudomonadaceae | *Cellvibrio* | 1 | 3 | 0 |
| Armatimonadetes |  |  |  | *Armatimonadetes* gp2 | 3 | 1 | 0 |
| Firmicutes | Negativicutes | Selenomonadales | Veillonellaceae | Sporomusa | 3 | 1 | 0 |
| Proteobacteria | Betaproteobacteria | Rhodocyclales | Rhodocyclaceae | *Shinella* | 2 | 0 | 2 |
| Proteobacteria | Alphaproteobacteria | Rhizobiales | Xanthobacteraceae | *Labrys* | 2 | 1 | 1 |
| Actinobacteria | Actinobacteria | Actinomycetales | Kineosporiaceae | *Kineosporia* | 1 | 2 | 1 |
| Bacteroidetes | Cytophagia | Cytophagales | Cytophagaceae | *Emticicia* | 0 | 0 | 4 |
| Proteobacteria | Alphaproteobacteria | Rhodobacterales | Rhodobacteraceae | *Gemmobacter* | 1 | 3 | 0 |
| Proteobacteria | Deltaproteobacteria | Myxococcales | Polyangiaceae | *Byssovorax* | 2 | 1 | 1 |
| Proteobacteria | Deltaproteobacteria | Myxococcales | Myxococcaceae | *Corallococcus* | 1 | 1 | 2 |
| Verrucomicrobia | Verrucomicrobiae | Verrucomicrobiales | Verrucomicrobiaceae | *Luteolibacter* | 2 | 2 | 0 |
| Actinobacteria | Actinobacteria | Actinomycetales | Nocardiaceae | *Rhodococcus* | 1 | 3 | 0 |
| Actinobacteria | Actinobacteria | Actinomycetales | Microbacteriaceae | *Leifsonia* | 1 | 2 | 1 |
| Hydrogenedentes |  |  |  | *Candidatus* Hydrogenedens | 4 | 0 | 0 |
| Proteobacteria | Alphaproteobacteria | Rhizobiales | Rhizobiales incertae sedis | *Bauldia* | 0 | 3 | 1 |
| Proteobacteria | Alphaproteobacteria | Rhizobiales | Rhodobiaceae | *Afifella* | 1 | 1 | 2 |
| Proteobacteria | Alphaproteobacteria | Rhizobiales | Beijerinckiaceae | *Chelatococcus* | 0 | 2 | 2 |
| Proteobacteria | Alphaproteobacteria | Sphingomonadales | Erythrobacteraceae | *Altererythrobacter* | 1 | 1 | 2 |
| Proteobacteria | Gammaproteobacteria | Xanthomonadales | Xanthomonadaceae | *Pseudoxanthomonas* | 0 | 0 | 4 |
| Actinobacteria | Actinobacteria | Actinomycetales | Intrasporangiaceae | *Terrabacter* | 1 | 2 | 1 |
| Actinobacteria | Actinobacteria | Actinomycetales | Thermomonosporaceae | *Actinoallomurus* | 0 | 4 | 0 |
| Proteobacteria | Betaproteobacteria | Rhodocyclales | Rhodocyclaceae | *Azonexus* | 1 | 1 | 1 |
| Firmicutes | Clostridia | Clostridiales | Clostridiaceae 1 | *Oxobacter* | 3 | 0 | 0 |
| Chlamydiae | Chlamydiia | Chlamydiales | Parachlamydiaceae | *Parachlamydia* | 0 | 3 | 0 |
| Proteobacteria | Betaproteobacteria | Burkholderiales | Comamonadaceae | *Ramlibacter* | 2 | 1 | 0 |
| Proteobacteria | Gammaproteobacteria | Pseudomonadales | Pseudomonadaceae | *Rhizobacter* | 0 | 3 | 0 |
| Proteobacteria | Betaproteobacteria | Rhodocyclales | Rhodocyclaceae | *Zoogloea* | 0 | 3 | 0 |
| Proteobacteria | Alphaproteobacteria | Rhodospirillales | Acetobacteraceae | *Acidiphilium* | 0 | 3 | 0 |
| Proteobacteria | Alphaproteobacteria | Sphingomonadales | Erythrobacteraceae | *Porphyrobacter* | 1 | 1 | 1 |
| Actinobacteria | Actinobacteria | Actinomycetales | Geodermatophilaceae | *Modestobacter* | 2 | 0 | 1 |
| Firmicutes | Bacilli | Bacillales | Planococcaceae | *Sporosarcina* | 0 | 1 | 2 |
| Bacteroidetes | Sphingobacteriia | Sphingobacteriales | Saprospiraceae | *Aureispira* | 3 | 0 | 0 |
| Bacteroidetes | Flavobacteriia | Flavobacteriales | Cryomorphaceae | *Wandonia* | 1 | 2 | 0 |
| Bacteroidetes | Cytophagia | Cytophagales | Cytophagaceae | *Hymenobacter* | 0 | 2 | 1 |
| Bacteroidetes | Cytophagia | Cytophagales | Cytophagaceae | *Arcicella* | 0 | 3 | 0 |
| Proteobacteria | Betaproteobacteria | Rhodocyclales | Rhodocyclaceae | *Uliginosibacterium* | 1 | 2 | 0 |
| Proteobacteria | Alphaproteobacteria | Rhodospirillales | Acetobacteraceae | *Craurococcus* | 0 | 1 | 2 |
| Proteobacteria | Gammaproteobacteria | Xanthomonadales | Sinobacteraceae | *Alkanibacter* | 0 | 3 | 0 |
| Verrucomicrobia | Verrucomicrobiae | Verrucomicrobiales | Verrucomicrobiaceae | *Prosthecobacter* | 2 | 1 | 0 |
| Verrucomicrobia | Verrucomicrobiae | Verrucomicrobiales | Verrucomicrobiaceae | *Verrucomicrobium* | 0 | 3 | 0 |
| Actinobacteria | Actinobacteria | Actinomycetales | Micromonosporaceae | *Virgisporangium* | 0 | 2 | 1 |
| Actinobacteria | Actinobacteria | Actinomycetales | Cryptosporangiaceae | *Cryptosporangium* | 0 | 1 | 2 |
| Actinobacteria | Actinobacteria | Actinomycetales | Jiangellaceae | *Jiangella* | 0 | 0 | 3 |
| Firmicutes | Clostridia | Clostridiales | Peptococcaceae 1 | *Desulfitobacterium* | 2 | 0 | 1 |
| Deinococcus-Thermus | Deinococci | Thermales | Thermaceae | *Meiothermus* | 0 | 3 | 0 |
| Firmicutes | Clostridia | Clostridiales | Clostridiales Incertae Sedis XI | *Sedimentibacter* | 2 | 0 | 0 |
| Proteobacteria | Alphaproteobacteria | Rhodospirillales | Acetobacteraceae | *Roseomonas* | 0 | 1 | 1 |
| Proteobacteria | Alphaproteobacteria | Rhodobacterales | Rhodobacteraceae | *Amaricoccus* | 1 | 0 | 1 |
| Bacteroidetes | Sphingobacteriia | Sphingobacteriales | Sphingobacteriaceae | *Solitalea* | 2 | 0 | 0 |
| Bacteroidetes | Cytophagia | Cytophagales | Cytophagaceae | *Spirosoma* | 1 | 1 | 0 |
| Actinobacteria | Actinobacteria | Actinomycetales | Cellulomonadaceae | *Cellulomonas* | 2 | 0 | 0 |
| Firmicutes | Negativicutes | Selenomonadales | Veillonellaceae | *Anaerospora* | 0 | 2 | 0 |
| Bacteroidetes | Sphingobacteriia | Sphingobacteriales | Chitinophagaceae | *Niabella* | 0 | 0 | 2 |
| Bacteroidetes | Flavobacteriia | Flavobacteriales | Flavobacteriaceae | *Moheibacter* | 0 | 0 | 2 |
| Proteobacteria | Betaproteobacteria | Burkholderiales | Burkholderiaceae | *Cupriavidus* | 0 | 1 | 1 |
| Proteobacteria | Alphaproteobacteria | Rhizobiales | Rhizobiaceae | *Kaistia* | 1 | 0 | 1 |
| Proteobacteria | Alphaproteobacteria | Rhodospirillales | Rhodospirillaceae | *Oceanibaculum* | 0 | 1 | 1 |
| Proteobacteria | Gammaproteobacteria | Xanthomonadales | Xanthomonadaceae | *Dokdonella* | 1 | 1 | 0 |
| Proteobacteria | Gammaproteobacteria | Enterobacteriales | Enterobacteriaceae | *Buttiauxella* | 0 | 2 | 0 |
| Proteobacteria | Gammaproteobacteria |  |  | *Candidatus* Carsonella | 0 | 1 | 1 |
| Proteobacteria | Deltaproteobacteria | Myxococcales | Phaselicystidaceae | *Phaselicystis* | 0 | 1 | 1 |
| Actinobacteria | Actinobacteria | Actinomycetales | Pseudonocardiaceae | *Lechevalieria* | 0 | 2 | 0 |
| Actinobacteria | Actinobacteria | Actinomycetales | Micromonosporaceae | *Actinoplanes* | 0 | 1 | 1 |
| Actinobacteria | Actinobacteria | Actinomycetales | Streptosporangiaceae | *Microbispora* | 0 | 2 | 0 |
| Actinobacteria | Actinobacteria | Actinomycetales | Streptosporangiaceae | *Sphaerisporangium* | 0 | 2 | 0 |
| Actinobacteria | Actinobacteria | Acidimicrobiales | Acidimicrobiaceae | *Ilumatobacter* | 0 | 1 | 1 |
| Firmicutes | Clostridia | Clostridiales | Clostridiales Incertae Sedis XVIII | *Symbiobacterium* | 0 | 0 | 2 |
| Firmicutes | Bacilli | Bacillales | Paenibacillaceae 1 | *Cohnella* | 2 | 0 | 0 |
| Firmicutes | Bacilli | Bacillales | Planococcaceae | *Lysinibacillus* | 0 | 1 | 1 |
| Firmicutes | Bacilli | Bacillales | Staphylococcaceae | *Staphylococcus* | 0 | 1 | 1 |
| Spirochaetes | Spirochaetia | Spirochaetales | Leptospiraceae | *Leptonema* | 1 | 1 | 0 |
| Proteobacteria | Betaproteobacteria | Rhodocyclales | Rhodocyclaceae | *Propionivibrio* | 1 | 0 | 0 |
| Proteobacteria | Deltaproteobacteria | Desulfuromonadales | Desulfuromonadaceae | *Desulfuromonas* | 1 | 0 | 0 |
| Firmicutes | Clostridia | Clostridiales | Gracilibacteraceae | *Lutispora* | 1 | 0 | 0 |
| Acidobacteria | Holophagae | Holophagales | Holophagaceae | *Holophaga* | 0 | 0 | 1 |
| Firmicutes | Clostridia | Clostridiales | Ruminococcaceae | *Saccharofermentans* | 1 | 0 | 0 |
| Firmicutes | Clostridia | Clostridiales | Ruminococcaceae | *Ethanoligenens* | 1 | 0 | 0 |
| Proteobacteria | Betaproteobacteria | Neisseriales | Neisseriaceae | *Vogesella* | 1 | 0 | 0 |
| Proteobacteria | Gammaproteobacteria | Xanthomonadales | Xanthomonadaceae | *Aspromonas* | 1 | 0 | 0 |
| Firmicutes | Bacilli | Bacillales | Planococcaceae | *Planococcaceae*  incertae sedis | 0 | 1 | 0 |
| Spirochaetes | Spirochaetia | Spirochaetales | Leptospiraceae | *Turneriella* | 1 | 0 | 0 |
| Proteobacteria | Betaproteobacteria | Burkholderiales | Oxalobacteraceae | *Naxibacter* | 1 | 0 | 0 |
| Proteobacteria | Betaproteobacteria | Hydrogenophilales | Hydrogenophilaceae | *Thiobacillus* | 0 | 1 | 0 |
| Proteobacteria | Gammaproteobacteria | Gammaproteobacteria incertae sedis |  | *Porticoccus* | 0 | 1 | 0 |
| Actinobacteria | Actinobacteria | Actinomycetales | Kineosporiaceae | *Angustibacter* | 1 | 0 | 0 |
| Firmicutes | Bacilli | Bacillales | Paenibacillaceae 1 | *Brevibacillus* | 0 | 0 | 1 |
| Bacteroidetes | Sphingobacteriia | Sphingobacteriales | Chitinophagaceae | *Hydrotalea* | 1 | 0 | 0 |
| Bacteroidetes | Sphingobacteriia | Sphingobacteriales | Sphingobacteriaceae | *Arcticibacter* | 0 | 0 | 1 |
| Bacteroidetes | Flavobacteriia | Flavobacteriales | Cryomorphaceae | *Crocinitomix* | 1 | 0 | 0 |
| Bacteroidetes | Cytophagia | Cytophagales | Cytophagaceae | *Runella* | 0 | 1 | 0 |
| Proteobacteria | Betaproteobacteria | Burkholderiales | Comamonadaceae | *Pseudorhodoferax* | 0 | 1 | 0 |
| Proteobacteria | Betaproteobacteria | Burkholderiales | Comamonadaceae | *Comamonas* | 0 | 1 | 0 |
| Proteobacteria | Betaproteobacteria | Burkholderiales | Oxalobacteraceae | *Herminiimonas* | 1 | 0 | 0 |
| Proteobacteria | Betaproteobacteria | Burkholderiales | Burkholderiaceae | *Ralstonia* | 0 | 0 | 1 |
| Proteobacteria | Alphaproteobacteria | Rhizobiales | Hyphomicrobiaceae | *Rhodomicrobium* | 0 | 1 | 0 |
| Proteobacteria | Alphaproteobacteria | Rhizobiales | Bradyrhizobiaceae | *Nitrobacter* | 1 | 0 | 0 |
| Proteobacteria | Alphaproteobacteria | Rhizobiales | Rhizobiaceae | *Ensifer* | 0 | 0 | 1 |
| Proteobacteria | Alphaproteobacteria | Rhizobiales | Beijerinckiaceae | *Beijerinckia* | 0 | 0 | 1 |
| Proteobacteria | Alphaproteobacteria | Rhizobiales | Aurantimonadaceae | *Aurantimonas* | 0 | 1 | 0 |
| Proteobacteria | Alphaproteobacteria | Rhodospirillales | Rhodospirillaceae | *Telmatospirillum* | 1 | 0 | 0 |
| Proteobacteria | Alphaproteobacteria | Rhodospirillales | Rhodospirillaceae | *Constrictibacter* | 0 | 0 | 1 |
| Proteobacteria | Alphaproteobacteria | Rhodospirillales | Acetobacteraceae | *Belnapia* | 0 | 1 | 0 |
| Proteobacteria | Alphaproteobacteria | Rhodospirillales | Acetobacteraceae | *Acidocella* | 0 | 0 | 1 |
| Proteobacteria | Alphaproteobacteria | Sphingomonadales | Sphingomonadaceae | *Sphingopyxis* | 0 | 0 | 1 |
| Proteobacteria | Alphaproteobacteria | Rhodobacterales | Rhodobacteraceae | *Rhodobacter* | 1 | 0 | 0 |
| Proteobacteria | Alphaproteobacteria | Rhodobacterales | Rhodobacteraceae | *Rubellimicrobium* | 0 | 0 | 1 |
| Proteobacteria | Gammaproteobacteria | Xanthomonadales | Xanthomonadaceae | *Xanthomonas* | 0 | 0 | 1 |
| Proteobacteria | Gammaproteobacteria | Xanthomonadales | Sinobacteraceae | *Hydrocarboniphaga* | 0 | 1 | 0 |
| Proteobacteria | Gammaproteobacteria | Legionellales | Coxiellaceae | *Coxiella* | 0 | 0 | 1 |
| Proteobacteria | Gammaproteobacteria | Pseudomonadales | Moraxellaceae | *Acinetobacter* | 0 | 1 | 0 |
| Proteobacteria | Gammaproteobacteria | Enterobacteriales | Enterobacteriaceae | *Pantoea* | 0 | 0 | 1 |
| Proteobacteria | Gammaproteobacteria | Enterobacteriales | Enterobacteriaceae | *Yersinia* | 0 | 1 | 0 |
| Proteobacteria | Deltaproteobacteria | Myxococcales | Nannocystaceae | *Nannocystis* | 0 | 0 | 1 |
| Proteobacteria | Deltaproteobacteria | Myxococcales | Polyangiaceae | *Jahnella* | 0 | 1 | 0 |
| Proteobacteria | Deltaproteobacteria | Myxococcales | Polyangiaceae | *Sorangium* | 0 | 0 | 1 |
| Proteobacteria | Deltaproteobacteria | Myxococcales | Myxococcaceae | *Myxococcus* | 1 | 0 | 0 |
| Acidobacteria | Acidobacteria Gp1 |  |  | *Edaphobacter* | 1 | 0 | 0 |
| Acidobacteria | Acidobacteria Gp1 |  |  | *Terriglobus* | 0 | 1 | 0 |
| Acidobacteria | Acidobacteria Gp16 |  |  | Gp16 | 1 | 0 | 0 |
| Acidobacteria | Acidobacteria Gp9 |  |  | Gp9 | 0 | 0 | 1 |
| Planctomycetes | Planctomycetia | Candidatus Brocadiales | Candidatus Brocadiaceae | *Candidatus* Kuenenia | 0 | 0 | 1 |
| Actinobacteria | Actinobacteria | Actinomycetales | Nocardiaceae | *Williamsia* | 1 | 0 | 0 |
| Actinobacteria | Actinobacteria | Actinomycetales | Nocardiaceae | *Smaragdicoccus* | 0 | 1 | 0 |
| Actinobacteria | Actinobacteria | Actinomycetales | Nocardioidaceae | *Flindersiella* | 0 | 0 | 1 |
| Actinobacteria | Actinobacteria | Actinomycetales | Micromonosporaceae | *Luedemannella* | 0 | 1 | 0 |
| Actinobacteria | Actinobacteria | Actinomycetales | Micromonosporaceae | *Rugosimonospora* | 0 | 1 | 0 |
| Actinobacteria | Actinobacteria | Actinomycetales | Acidothermaceae | *Acidothermus* | 0 | 0 | 1 |
| Actinobacteria | Actinobacteria | Actinomycetales | Microbacteriaceae | *Agrococcus* | 1 | 0 | 0 |
| Actinobacteria | Actinobacteria | Actinomycetales | Microbacteriaceae | *Subtercola* | 0 | 1 | 0 |
| Actinobacteria | Actinobacteria | Actinomycetales | Sanguibacteraceae | *Sanguibacter* | 1 | 0 | 0 |
| Actinobacteria | Actinobacteria | Actinomycetales | Bogoriellaceae | *Georgenia* | 0 | 0 | 1 |
| Actinobacteria | Actinobacteria | Actinomycetales | Promicromonosporaceae | *Promicromonospora* | 0 | 0 | 1 |
| Actinobacteria | Actinobacteria | Actinomycetales | Thermomonosporaceae | *Actinocorallia* | 0 | 1 | 0 |
| Firmicutes | Clostridia | Clostridiales | Clostridiales Incertae Sedis XI | *Anaerococcus* | 1 | 0 | 0 |
| Firmicutes | Clostridia | Clostridiales |  | *Natranaerovirga* | 1 | 0 | 0 |
| Firmicutes | Bacilli | Bacillales | Bacillaceae 1 | *Falsibacillus* | 1 | 0 | 0 |
| Firmicutes | Bacilli | Bacillales | Planococcaceae | *Caryophanon* | 0 | 1 | 0 |
| Firmicutes | Bacilli | Bacillales | Bacillales Incertae Sedis XII | *Exiguobacterium* | 1 | 0 | 0 |
| Firmicutes | Bacilli | Lactobacillales | Lactobacillaceae | *Lactobacillus* | 0 | 1 | 0 |
| Firmicutes | Erysipelotrichia | Erysipelotrichales | Erysipelotrichaceae | *Turicibacter* | 1 | 0 | 0 |
| Spirochaetes | Spirochaetia | Spirochaetales | Leptospiraceae | *Leptospira* | 0 | 1 | 0 |
| Chloroflexi | Caldilineae | Caldilineales | Caldilineaceae | *Caldilinea* | 1 | 0 | 0 |
| Chloroflexi | Chloroflexia | Chloroflexales | Oscillochloridaceae | *Oscillochloris* | 0 | 1 | 0 |
| Cyanobacteria/Chloroplast | Cyanobacteria |  | Family I | GpI | 0 | 1 | 2 |
| Elusimicrobia | Endomicrobia |  |  | *Candidatus* Endomicrobium | 0 | 0 | 1 |
| Deinococcus-Thermus | Deinococci | Deinococcales | Deinococcaceae | *Deinococcus* | 0 | 1 | 0 |
